# Musical expertise and foreign speech perception

**DOI:** 10.3389/fnsys.2013.00084

**Published:** 2013-11-14

**Authors:** Eduardo Martínez-Montes, Heivet Hernández-Pérez, Julie Chobert, Lisbet Morgado-Rodríguez, Carlos Suárez-Murias, Pedro A. Valdés-Sosa, Mireille Besson

**Affiliations:** ^1^Cuban Neuroscience CenterHavana, Cuba; ^2^Laboratoire de Neuroscience Cognitive, CNRS-Aix Marseille UniversitéMarseille, France

**Keywords:** musical expertise, auditory perception, speech perception, foreign language, pitch, duration, Voice Onset Time, Mismatch Negativity

## Abstract

The aim of this experiment was to investigate the influence of musical expertise on the automatic perception of foreign syllables and harmonic sounds. Participants were Cuban students with high level of expertise in music or in visual arts and with the same level of general education and socio-economic background. We used a multi-feature Mismatch Negativity (MMN) design with sequences of either syllables in Mandarin Chinese or harmonic sounds, both comprising deviants in pitch contour, duration and Voice Onset Time (VOT) or equivalent that were either far from (Large deviants) or close to (Small deviants) the standard. For both Mandarin syllables and harmonic sounds, results were clear-cut in showing larger MMNs to pitch contour deviants in musicians than in visual artists. Results were less clear for duration and VOT deviants, possibly because of the specific characteristics of the stimuli. Results are interpreted as reflecting similar processing of pitch contour in speech and non-speech sounds. The implications of these results for understanding the influence of intense musical training from childhood to adulthood and of genetic predispositions for music on foreign language perception are discussed.

## Introduction

Normally-developing infants can learn any of the world languages as nicely stated by Patricia Kuhl: “children are born citizens of the world” (Kuhl, 2002). Unfortunately, this wonderful ability seems to quickly disappear as shown in an elegant experiment by Cheour et al. (1998). These authors used the well-known Mismatch Negativity (MMN) paradigm (Näätänen et al., 1978) to test for the idea of a sensitive period in phoneme perception. Results showed that by one year of age, Finnish phonemes had acquired a special status for Finnish children compared to a phoneme (in Estonian) that did not belong to the Finnish phoneme repertory. These results are clear evidence in favor of an early critical period for phoneme acquisition.

Nevertheless, humans can learn new languages at any time in life even if factors like the starting age of learning (e.g., Flege et al., 1995; Birdsong, 2005, 2006), the amount of knowledge in the native language (e.g., Flege and MacKay, 2004) and the proximity between native (L1) and second language (L2) phonetic inventory (e.g., Flege, 1995; Best et al., 2001) are known to influence learning efficiency (e.g., Golestani and Zatorre, 2009), together with extra-linguistic factors such as motivation (Moyer, 1999), working memory (Miyake and Friedman, 1998; Majerus et al., 2008) and attention control (Segalowitz, 1997; Guion and Pederson, 2007).

Of most interest for our concerns, musical expertise has also been shown to influence foreign language perception and production (Chobert and Besson, 2013). Slevc and Miyake (2006) tested Japanese adults immersed in their L2 (English) after the age of 11 and controlled for the age of first L2 exposure, working memory and level of L2 use. Results of correlation analyses showed that musical abilities were predictive of phonological abilities tested through the perception and production of the English /r/-/l/ contrast that does not belong to the Japanese phoneme repertory. Tervaniemi and collaborators (Milovanov et al., 2008, 2010) also reported that Finnish children and young adults with advanced musical skills had better English pronunciation abilities than those with less-advanced musical skills. Studying prosody perception and recording ERPs together with behavior, Marques et al. (2007) showed that French adult musicians perceived subtle pitch changes at the end of Portuguese sentences, a language that they did not understand, better than French non-musicians. The onset latency of the late positivity to pitch deviants was 300 ms earlier in musicians than in non-musicians. Similar results were reported by Deguchi et al. (2012) with Italian musicians and non-musicians presented with pitch changes in syntactically correct or jabberwocky Italian and French sentences. Taken together, these results suggest an interconnection between musical expertise and foreign language perception and production.

In most of the experiments aimed at testing the influence of musical expertise on foreign language perception, lexical tones have been used as stimuli presented to native and non-native tone language speakers (e.g., Gottfried and Riester, 2000; Alexander et al., 2005; Delogu et al., 2006, 2010; Gottfried, 2007; Wong et al., 2007; Lee and Hung, 2008; Marie et al., 2011a). At the behavioral level, Gottfried and Riester (2000) and Gottfried (2007) showed that English musicians unfamiliar with tone languages identified the four Mandarin tones better than non-musicians. Moreover, Lee and Hung (2008) reported that English musicians were more accurate than non-musicians in identifying intact syllables produced on the four Mandarin tones among syllables modified in pitch height or pitch contour.

At the subcortical level, Wong et al. (2007) recorded the brainstem Frequency Following Response (FFR) to the contour patterns of Mandarin tones in English amateur musicians and non-musicians who were unfamiliar with tone languages. They reported higher quality of linguistic pitch encoding in the auditory brainstem responses of musicians compared to non-musicians. More recently, Bidelman et al. (2011) presented iterated rippled noise homologue of a lexical tone to English amateur musicians and non-musicians and to Mandarin Chinese speakers. Pitch-tracking accuracy and the strength of the FFR were larger in musicians and in Chinese speakers than in English non-musicians. Finally, Chandrasekaran et al. (2012) demonstrated that the relationship between the efficiency of inferior colliculus pitch representations (assessed by fMRI-Adaptation) and the quality of neural pitch pattern representations (assessed by auditory brainstem recordings) was stronger in musicians than in non-musicians.

At the cortical level, Chandrasekaran et al. (2009) showed that deviants in pitch contour homologous to Mandarin tones elicited larger MMNs in English musicians than in English non-musicians, thereby showing increased automatic processing of pitch variations in both music and speech in the musician group. Using an active discrimination task on sequences of Mandarin Chinese monosyllabic words, Marie et al. (2011a) recorded behavioral measures and ERPs from French musicians and non-musicians, unfamiliar with tone languages. Musicians detected both supra-segmental tonal and segmental (consonant, vowel) variations more accurately than non-musicians. Moreover, tonal variations were categorized faster by musicians than by non-musicians, as reflected by the shorter latency of the N2/N3 components (see also Fujioka et al., 2006; Moreno et al., 2009). Finally, the decision that tone and/or segmental variations were different was associated with larger P3b components (e.g., Duncan-Johnson and Donchin, 1977; Picton, 1992) in musicians than in non-musicians.

Taken together, studies of lexical tone perception by non-native listeners showed that musicians discriminated and/or identified segmental and supra-segmental linguistic contrasts in a foreign language better than non-musicians. Results also revealed more reliable encoding of linguistic pitch patterns at the subcortical level as well as enhanced MMNs to pitch contour deviants and enhanced discrimination and decision-related ERP components at the cortical level in musicians compared to non-musicians. These results demonstrate that long-term musical training not only facilitates the processing of unattended and attended harmonic and musical sounds but also impacts on the processing of speech sounds. These findings have been taken as evidence that some aspects of music and speech involve common processing mechanisms and transfer effects (see Kraus and Chandrasekaran, 2010 and Besson et al., 2011, for reviews).

While several studies have examined the influence of musical expertise on non-native tone perception, only one study has, to our knowledge, examined the influence of musical expertise on non-native vowel duration, a phonemic contrast that is linguistically relevant in quantity languages such as Finnish or Japanese. Sadakata and Sekiayama (2011) recently examined categorical perception of both supra-segmental moraic features in Japanese and segmental vowels variations in Dutch by presenting Dutch and Japanese musicians and non-musicians with discrimination and identification tests. The mora is defined as a perceptual temporal unit and is used by Japanese listeners to segment speech signals (Cutler and Otake, 1994). Results of the same/different discrimination task with pairs of Japanese (e.g., kanyo-kannyo) and Dutch words (e.g., kuch-kech), differing in morae or vowel respectively, showed that musicians, Dutch and Japanese, outperformed non-musicians in the discrimination of supra-segmental and segmental variations in their own language, as well as in the foreign language. Moreover, after learning, identification performance of moraic feature (in stop Japanese contrast) was higher in musicians (Japanese and Dutch) than in non-musicians. These results are important because they demonstrate that musical expertise not only influences phoneme perception and discrimination but also categorical perception. As such, they raise the interesting possibility that musical expertise enhances the ability to build reliable abstract phonological representations (e.g., Slevc and Miyake, 2006; Degé and Schwarzer, 2011; Ott et al., 2011).

Based on these results, the overall aim of the present study was to use the MMN to determine whether musical expertise influences the perception of non-native supra-segmental and segmental speech variations when participants are not required to focus attention on the phonemic contrasts of interest. We examined three types of phonemic contrasts: pitch contour, vowel duration and Voice Onset Time (VOT). VOT is a phonological parameter acoustically defined as the interval between the noise burst produced at consonant release and the onset of the waveform periodicity associated with vocal cord vibration (Lisker and Abramson, 1967). Changes in VOT typically allow one to perceive stop consonants as voiced (e.g., /b/) or voiceless (e.g., /p/). To our knowledge, no studies have yet investigated the automatic processing of vowel duration and VOT contrasts in a language unknown for the participants. However, two recent studies have investigated the automatic processing of these phonemic contrasts in the participants' native language. Chobert et al. (2011) tested 9-year-old children and found larger MMNs to vowel duration and VOT deviants in musician compared to non-musician children. Very recently, Kühnis et al. (2013) reported enhanced MMNs to native contrasts of vowel frequency (fundamental frequency and second formant transition), vowel duration and VOT deviants in musician compared to non-musician adults. Thus, it was of interest to determine whether similar results would be obtained with non-native phonemic contrasts.

To this aim we used a multi-feature MMN design (Näätänen et al., 2004) with Mandarin Chinese syllables presented to Cuban musicians and visual artists. The syllable “Cha” was used as standard and deviant syllables were either close to or far from the standard (Small and Large deviants) on three dimensions: pitch contour, vowel duration and VOT. Based on the results summarized above (Chandrasekaran et al., 2009; Chobert et al., 2011; Kühnis et al., 2013), we hypothesized that musicians should be more sensitive than non-musicians to spectrally (pitch contour) and temporally (vowel duration and VOT) deviant Mandarin Chinese syllables, even if these syllables were unfamiliar to all participants. Moreover, based on previous results (e.g., Chobert et al., 2011; Marie et al., 2012), we also expected larger differences between musicians and visual artists for Small deviants, that are difficult to perceive, than for Large deviants, that are easy to perceive and can be detected by both groups of participants.

In addition, we controlled that musicians were more sensitive than non-musicians to manipulations of harmonic sounds similar to those created for Mandarin Chinese syllables. The note “Mi3” played on a clarinet was used as standard with Small and Large deviant sounds/notes on three dimensions: pitch contour, duration and an equivalent of VOT (see Methods). We hypothesized that Cuban musicians should be more sensitive than Cuban visual artists to the different manipulations of the harmonic sounds, again with larger between-group differences for Small (difficult to detect) than for Large deviants (easy to detect).

In sum, the originality of the present study was to compare spectral and temporal automatic processing for both syllables and harmonic sounds within the same participants, to use spectral manipulations of pitch contour that are linguistically relevant in Mandarin Chinese and to create manipulations of harmonic sounds as similar as possible from the linguistic sounds. Moreover, because this study was conducted in Cuba, all participants had very homogenous socio-economic background and level of education.

## Methods

### Participants

Twenty six musicians and twenty six visual artists from the “Instituto Superior de Arte” (ISA; Superior Institute of Art) participated in this experiment that lasted for about 2 h. All subjects were native speakers of Spanish, with no experience of tone languages and without hearing or neurological disorders. None of the visual artists had any formal musical training other than that provided in elementary school and none of them played a musical instrument. Musicians started musical training around the age of 7 and had 16 ± 4 years of musical training on average at the time of testing. They played different instruments as detailed in Table 1. Visual artists started training in painting and/or sculpturing around 14 and had 7 ± 3 years of training on average. Four musicians and four visual artists were not included in the analyses because of too many artifact-contaminated trials in their EEG recordings. The final groups comprised 22 musicians (mean age 23.4 ± 3.6, 11 women; 17 right-handed, 2 left-handed and 3 ambidextrous) and 22 visual artists (mean age 23.4 ± 2.0, 8 women; 19 right-handed, 2 left-handed and 1 ambidextrous). All participants gave their informed consent to participate in the experiment that was conducted according to the ethical guidelines of the Cuban Neuroscience Center.

**Table 1:** **List of instruments or abilities of the musicians participating in the study**.

**Instruments or abilities**	**No. of Subjects**
Piano	6
Violin	4
Orchestral conducting	4
Composition	4
Fagot	1
Tres	1
Lyric singing	1
Percussion	1

### Stimuli

Linguistic stimuli were built from the natural syllables “Cha” (Tone 1), “Chá” (Tone 2), and “Zha” spoken by a Mandarin Chinese native speaker by using the Praat software (Boersma and Weenink, 2009). All stimuli had a Consonant-Vowel structure and a mean intensity of 70 dB SPL (except for intensity deviants^1^). For all stimuli (linguistic and non-linguistic), intensity was calibrated in dB SPL using a Brüel & Kjaer sound level meter (Investigator 2250 with microphone type 4189). The standard stimulus “Cha” comprised the consonant “Ch” (105 ms in duration) and the vowel “a” (Tone 1; 260 ms in duration) with a total duration of 365 ms and a fundamental frequency (F0) of 164.8 Hz (defined from the first to the last pulse of the vowel).

Deviants were chosen based upon the results of a pilot study with 12 Chinese native speakers, 12 Cuban musicians and 13 Cuban non-musicians. We first digitally created continuous changes in F0, vowel duration, consonant duration (VOT) and average vowel intensity from the standard syllable “Cha”. For pitch deviants, the F0 was increased using a sigmoid function extracted from the original recording of “Chá” (Tone 2), going from standard F0 (164.8 Hz) in the first pulse of the vowel to different ending frequencies in the last pulse of the vowel (resulting in frequency changes of 5, 10, 20, 30, 50, 80, 100, 200, 300, 400, and 500 cents of semitones). For Duration deviants, the duration of the vowel was shortened by re-synthesis in steps of 10 ms, from 260 ms (standard) to 140 ms. VOT deviants were built by shortening the consonants of “Cha” and “Zha” by 10 ms (using the nearest zero-crossing point to avoid high frequency artifacts). A continuum was built from the standard “Cha” to “Zha.” Finally, for intensity deviants, intensity was decreased in steps of 2 db, from 70 db (standard) to 62 db.

Pilot participants listened to 48 pair of sounds (standard-deviant stimuli) and they had to decide whether they were same or different. Based on these results, we selected the large deviant for each condition (frequency, duration, VOT and intensity) as the one similarly detected by all groups. By contrast, small deviants were the ones better detected by musicians and Chinese participants than by non-musicians. In the case of VOT, the large deviant was the unmodified “Zha” syllable (across phonetic category). The small deviant was a small change to the consonant “Ch” that sounded more similar to “Zh” but was still recognized as “Ch” by all groups in the pilot study.

Non-linguistic stimuli (harmonic sounds) were built by using procedures similar to those used for linguistic sounds. All stimuli were high-quality recordings of natural clarinet sounds with a mean intensity of 70 dB SPL (except for intensity deviants^1^). The standard sound had an F0 of 164.8 Hz (Mi3) and a total duration of 260 ms. Continuous changes in F0 (following the same sigmoid function as in syllables), duration and intensity were built in the same way as for the linguistic stimuli. For VOT deviants, the first part of the sound was removed (zeroed) in steps of 5 ms in order to obtain a different temporal relationship between the low frequency components of the clarinet timber (starting at the beginning) and the higher frequency components (starting around 65 ms). This procedure is similar to the one used in previous studies (e.g., Chobert et al., 2011) to convert a “Ba” into a “Pa.” The total duration of the sounds was kept to 260 ms to ensure that all stimuli (standard and deviants) were synchronized in time to the first pulse. Pilot participants were presented with 48 pairs in a same/different task as described above for syllables. In each condition (frequency, duration, VOT and intensity), we selected a large deviant that was similarly detected by all groups, while the small deviant was better detected by musicians than by non-musicians.

**Figure 1:**
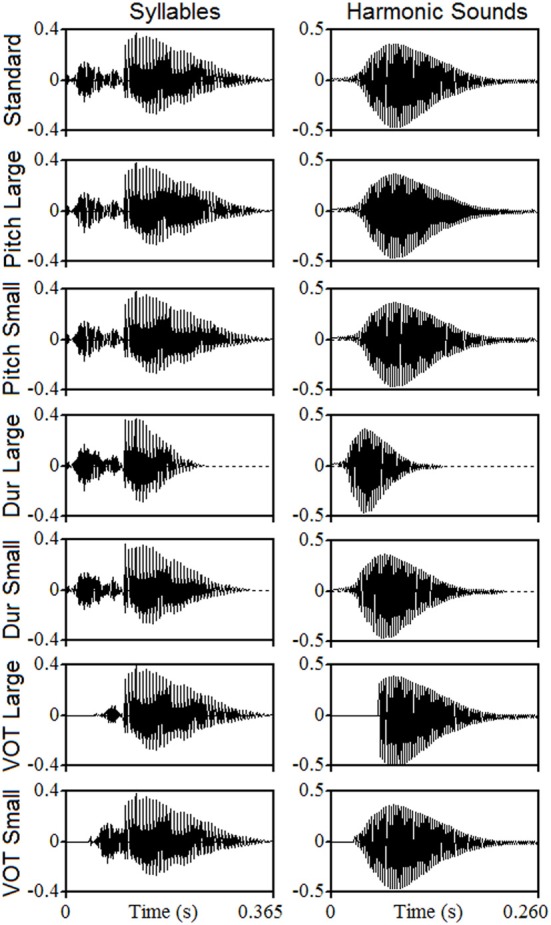
**Waveforms of the auditory stimuli.** The first row shows the two standard stimuli: the Mandarin “Cha” syllable (left column) and the note “Mi3” of a clarinet harmonic sound (right column). The other rows show the different deviants. Waveforms are temporally synchronized to the first pulse of the harmonic sound and of the vowel.

Figure 1 illustrates the sound waveforms, whose acoustic properties are summarized in Table 2. In both syllables and harmonic sounds and for Large pitch contour deviants, the F0 increased from 164.8 to 185.0 Hz (a continuous increase of 2 semitones that is 20.2 Hz, 11% increase). For Small pitch deviants, the F0 increased from 164.8 to 169.6 Hz (50 cents of a tone that is 4.8 Hz, 2.9% increase) in harmonic sounds and from 164.8 to 168.7 Hz (40 cents of a tone that is 3.9 Hz, 2.4% increase) in syllables. For both type of stimuli, Large duration deviants were 120 ms shorter than the Standard (46.2% decrease) and Small duration deviants were 40 ms shorter than the Standard (15.4% decrease). For linguistic stimuli, the syllable “Zha” was used as Large VOT deviant. The Small VOT deviant comprised the first 60 ms of the consonant “Ch” joined to the vowel of the standard. In both cases, a period of silence was added at the beginning to keep a total duration of 365 ms (same as standard). For harmonic sounds, the Large and Small VOT deviants were built by zeroing the first 60 and 30 ms of the sound, respectively (using the nearest zero-crossing point to avoid artifacts in both cases).

**Table 2:** **Summary of the acoustic properties of auditory stimuli**.

**Stimulus**	**Mandarin syllables**			**Harmonic sounds**		
	**Pitch Contour (Hz)**	**Duration (ms)**	**VOT (ms)**	**Pitch Contour (Hz)**	**Duration Vowel (ms)**	**VOT (consonant)**
Standard	164.8–164.8	260	CH (105 ms)	164.8–164.8	260	0
Pitch large	164.8–185.0	–	–	164.8–185.0	–	–
Pitch small	164.8–168.7	–	–	164.8–169.6	–	–
Duration large	–	140	–	–	140	–
Duration small	–	220	–	–	220	–
VOT large	–	–	ZH (50 ms)	–	–	60
VOT small	–	–	CH (60 ms)	–	–	30

^*^VOT for harmonic sounds is defined as the time set to zero from the beginning of the stimuli. For Syllables it represents the length of the first part of the consonant used. The dash (−) represents the same value as the standard.

### Mmn experiment procedure

The EEG was recorded while participants sat in a comfortable chair and watched a silent subtitled movie of their choice displayed on a screen at one meter distance. Participants were asked to watch the movie without paying attention to the sounds that were presented through headphones.

Syllables and harmonic sounds were presented in two separate blocks that lasted for 12.2 min each. Pitch, duration, VOT and intensity deviants were presented in a balanced pseudorandom order, always including one or two standards sounds between deviants. Five different sequences were created with a Stimulus Onset Asynchrony (SOA) of 700 ms and with a total of 1200 stimuli: 504 deviants (72 for each of the 7 deviant types; 6% probability) and 696 standards plus 15 standards at the beginning of the sequence. Sequences were balanced between subjects. Moreover, half of the participants started with the syllable sequences and the other half with the harmonic sound sequences. Participants were asked questions at the end of the experiment to ensure they had paid attention to the movie.

### Erp recordings

The EEG was continuously recorded from 32 Biosemi pin-type active electrodes (Amsterdam University), mounted on an elastic head cap, and located at standard left and right hemisphere positions over frontal, central, parietal, occipital, and temporal areas (International 10/20 system sites: Fz, Cz, Pz, Oz, Fp1, Fp2, AF3, AF4, F3, F4, C3, C4, P3, P4, P7, P8, O1, O2, F7, F8, T7, T8, Fc5, Fc1, Fc2, Fc6, Cp5, Cp1, Cp2, Cp6, PO3, PO4). Moreover, to detect horizontal eye movements and blinks, the Electro-oculogram (EOG) was recorded from Flat-type active electrodes placed 1 cm to the left and right of the external canthi, and from an electrode beneath the right eye. Two additional electrodes were placed on the left and right mastoids. EEG was recorded at a sampling rate of 512 Hz using Biosemi amplifiers. The EEG was re-referenced offline to the algebraic average of the left and right mastoids and filtered with a band-pass of 1–30 Hz (12 db/oct). Impedances of the electrodes never exceeded 5 kΩ. Data were segmented in single trials of 800 ms starting 100 ms before stimuli onset and were analyzed using the BrainVision Analyzer software (Brain Products, Munich). Trials containing ocular artifacts (75 μV threshold on vertical and horizontal EOG) and movement artifacts (75 μV threshold on all channels) were excluded from the averaged ERP waveforms. On average, the number of rejected trials for each deviant stimulus was less than 15% of the total number of trials.

### Data analyses

Artifact-free ERP trials (mastoid referenced) were averaged for each subject and for each experimental condition. Difference waveforms were obtained by subtracting the ERPs elicited by the Standard stimuli from those elicited by each deviant stimulus. The MMN was also computed by using the nose reference to ensure the typical MMN inversion between Fz/Cz and the mastoid electrodes (for review, see Näätänen et al., 2007). However, because mastoid-referenced averages typically show a better signal-to-noise ratio than the nose-referenced averages (e.g., Kujala et al., 2007), the former were used to quantify MMN amplitude.

For each condition, *Z*-tests were performed to compare each time point of the grand average traces of each group (musicians, MUS and Visual Artists, VA) against zero. The significant points were selected as those with Z-statistics higher than the threshold corresponding to a corrected significance level of 0.05 (using Bonferroni correction). Mean MMN amplitude were measured in a 50 ms time window, centered at the peak negative value. The same procedure was used for the other two components: the early negativity to Large duration and VOT deviants (peaking around 100–120 ms) and the P3a to Large duration deviants (peaking around 500 ms). Maximum MMN amplitude developed between 200 and 400 ms for both pitch and duration deviants in harmonic sounds, and slightly later in syllables. Maximum MMN amplitudes for VOT deviants were between 150 and 250 ms in both harmonic sounds and Mandarin syllables (see next section).

Given the different nature of the stimuli used for Pitch, Duration and VOT, and because the MMNs were largest at frontal electrodes, Three-Way ANOVAs were conducted at frontal sites for each Dimension separately that included Group (MUS vs. VA) as a between-subject factor and Deviance size (Small vs. Large) and Laterality (Left vs. Midline vs. Right) as within-subject factors. As the Group by Laterality interaction was not significant for syllables or for harmonic sounds, these results are not reported further. Results of exploratory analyses (ANOVAs) conducted for large and Small deviants separately are also reported when they allow a better understanding of the effects of interest.

## Results

### Mmn in each condition against zero

Results of two-tailed *Z*-tests vs. 0 using the Bonferroni correction across time (alpha = 0.05) showed that the MMNs to large deviants, whether in syllables or in harmonic sounds, were always significantly different from zero in both groups but the latency band within which these differences were significant varied. The MMNs to small pitch deviants in harmonic sounds were significantly different from zero in both groups but only for musicians for syllabic pitch. The MMNs to small duration deviants were only significantly different from zero for musicians and for harmonic sounds. Finally, the MMNs to small VOT (or equivalent) deviants were not significantly different from zero either for syllables or for harmonic sounds.

### Mmn differences between groups for the three types of deviants

**Figure 2:**
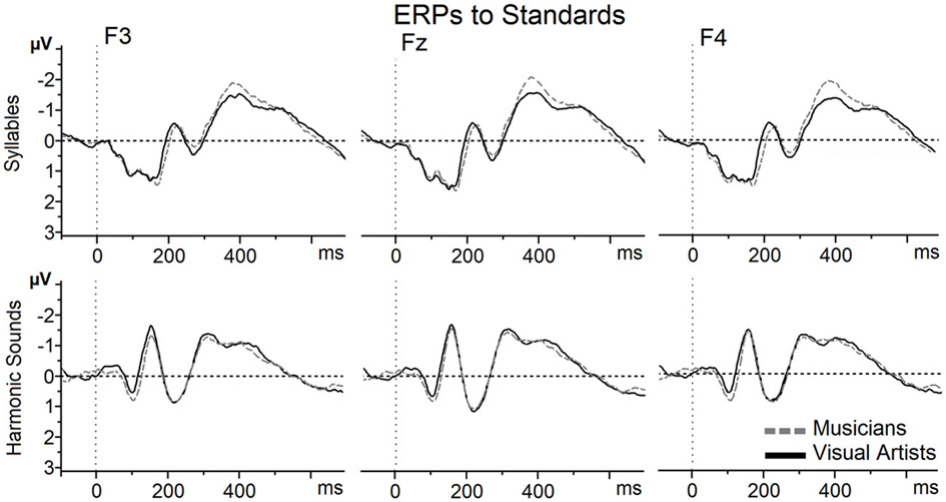
**ERPs to standards, Mandarin syllable and harmonic sounds, recorded at Frontal sites (F3, Fz, and F4) are overlapped for musicians (gray dashed line) and visual artists (black solid line).** On this and subsequent figures, time in milliseconds is in abscissa and the amplitude of the effects in microvolts is in ordinate. Time zero corresponds to sound onset and negativity is up.

In order to control that the MMN differences reported below are due to the processing of the deviants rather than to the standards, *t*-tests were first computed between both groups (Bonferroni corrected) on the standards in each condition. As illustrated on Figure 2, the ERPs to both syllables and harmonic sounds very well overlap in the two groups and results revealed no significant differences. Thus, the between-groups differences that we report below for the MMN are more likely linked to the deviants than to the standards.

#### Pitch contour deviants

**Figure 3:**
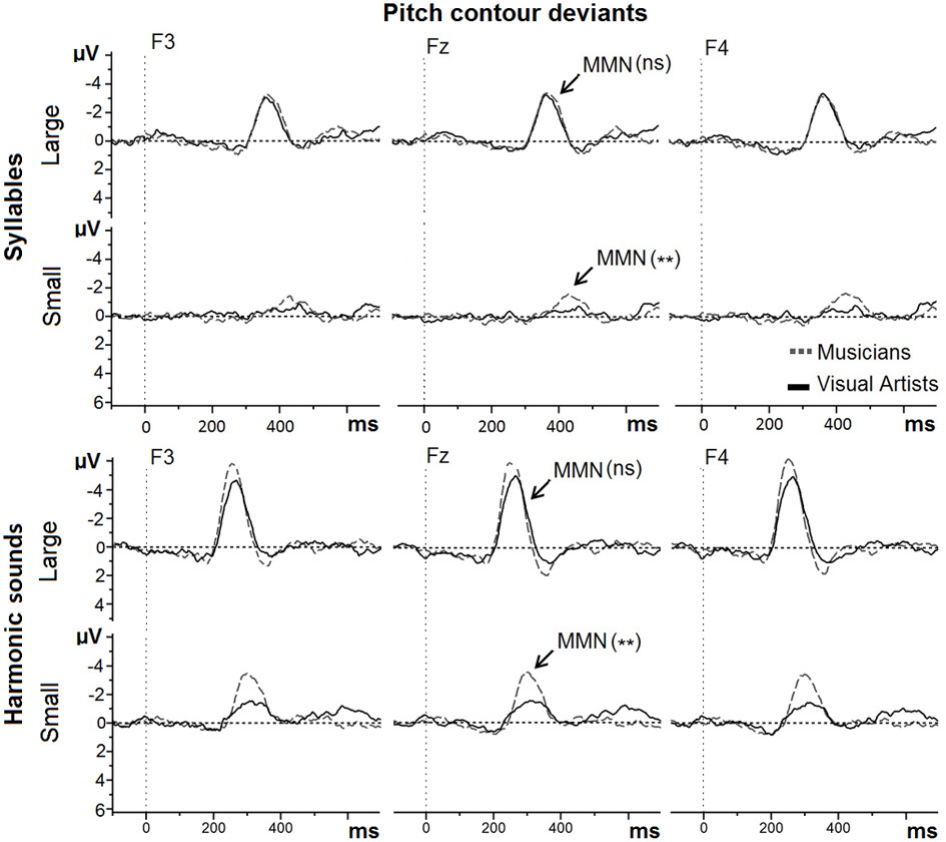
**MMNs to Large and Small pitch contour deviants in Mandarin syllables and in harmonic sounds recorded at Frontal sites (F3, Fz, and F4) are overlapped for musicians (gray dashed line) and visual artists (black solid line).** On this and subsequent figures: ns means “not significant”; one (^*^) and two stars (^**^) represent significance with *p* < 0.05 and *p* < 0.01, respectively.

For pitch contour deviants in Mandarin syllables (see Figure 3, upper row), the MMN was marginally larger in MUS (−2.73 μV) than in VA [−2.06 μV; main effect of Group: *F*_(1, 42)_ = 3.18, *p* = 0.08]. Large deviants (−3.42 μV) elicited larger MMNs than Small deviants [−1.36 μV; main effect of Deviance size: *F*_(1, 42)_ = 40.59, *p* < 0.001]. The Group by Deviance size interaction was significant [*F*_(1, 42)_ = 9.70, *p* < 0.001]. Separate analyses for Large and Small deviants revealed that while the MMNs to Large deviants were not significantly different between MUS (−3.58 μV) and VA (−3.28 μV; *F* < 1), the MMNs to Small deviants were larger in MUS (−1.88 μV) than in VA [−0.85 μV; *F*_(1, 42)_ = 11.41, *p* < 0.002].

For pitch contour deviants in harmonic sounds (see Figure 3, lower row), the MMN was larger in MUS (−5.00 μV) than in VA [−3.40 μV; main effect of Group: *F*_(1, 42)_ = 6.61, *p* < 0.02]. Large deviants (−5.53 μV) elicited larger MMNs than Small deviants [−2.87 μV; main effect of Deviance size: *F*_(1, 42)_ = 94.88, *p* < 0.001]. The Group by Deviance size interaction was not significant [*F*_(1, 42)_ = 2.60, *p* > 0.10] but subsequent exploratory analyses revealed that while the MMNs to Large harmonic pitch deviants were not significantly different between MUS (−6.11 μV) and VA (−4.94 μV; [*F*_(1, 42)_ = 2.41, *p* > 0.10], the MMNs to Small duration deviants were larger in MUS (−3.90 μV) than in VA [−1.85 μV; *F*_(1, 42)_ = 11.52, *p* < 0.001].

#### Duration deviants

**Figure 4:**
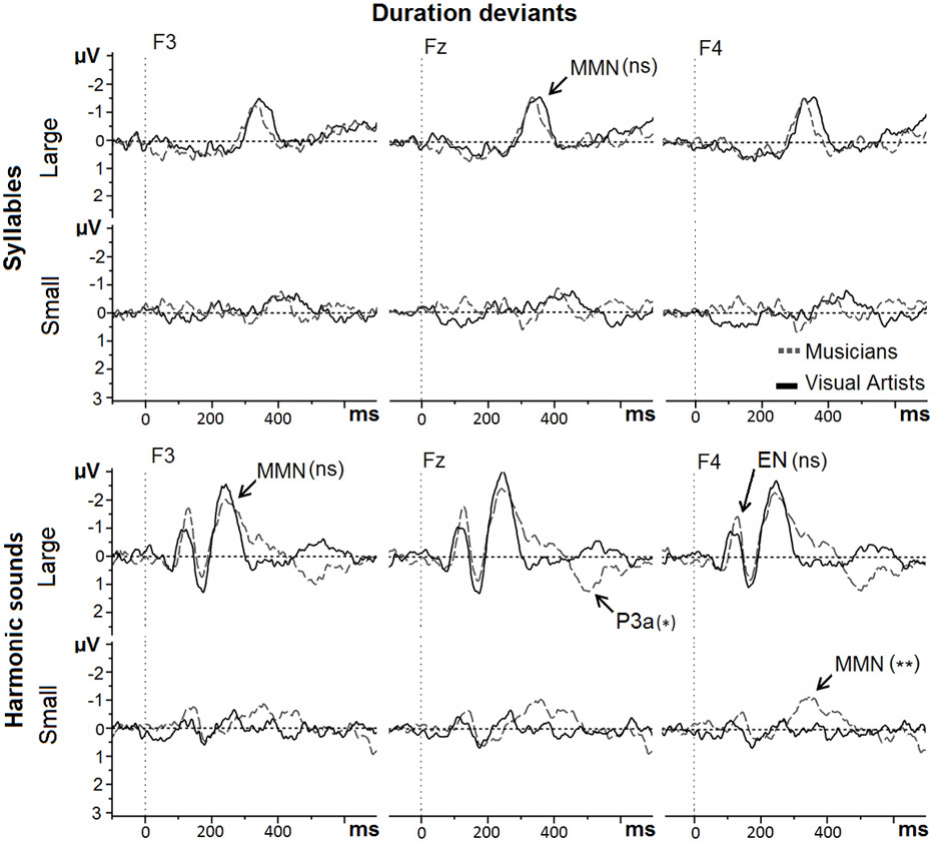
**MMNs to large and small duration deviants in Mandarin syllables and in harmonic sounds recorded at Frontal sites (F3, Fz, and F4) are overlapped for musicians (gray dashed line) and visual artists (black solid line)**.

For duration deviants in Mandarin syllables (see Figure 4, upper row), the main effect of Group was not significant (MUS = −1.62 μV; VA = −1.41 μV; *F* < 1). Large deviants (−1.89 μV) elicited larger MMNs than Small deviants [−1.14 μV; main effect of Deviance size: *F*_(1, 42)_ = 12.79, *p* < 0.001]. The Group by Deviance size interaction was not significant [*F*_(1, 42)_ = 2.54, *p* > 0.10]: MMNs to Large and Small Duration deviants were not significantly different between groups.

For duration deviants in harmonic sounds (see Figure 4, lower row), the main effect of Group was marginally significant [MUS = −2.33 μV; VA = −1.74 μV; *F*_(1, 42)_ = 3.23, *p* < 0.08]. Large deviants (−2.87 μV) elicited larger MMNs than Small deviants [−1.20 μV; main effect of Deviance size: *F*_(1, 42)_ = 41.88, *p* < 0.001]. The Group by Deviance size interaction was marginally significant [*F*_(1, 42)_ = 3.04, *p* = 0.08]. Subsequent exploratory analyses revealed that while the MMNs to Large duration deviants were not significantly different between MUS (−2.94 μV) and VA (−2.80 μV; *F* < 1), the MMNs to Small duration deviants were larger in MUS (−1.72 μV) than in VA [−0.68 μV; *F*_(1, 42)_ = 7.45, *p* < 0.01] thereby explaining that the main effect of Group was marginally significant.

For Large duration deviants, the early negativity (around 120 ms) was not significantly different between the two groups (*F* < 1) but the P3a component (around 500 ms) was significantly larger in MUS (0.81 μV) than in VA [−0.27 μV; main effect of Group: *F*_(1, 42)_ = 5.56, *p* < 0.03].

#### Vot (or equivalent) deviants

**Figure 5:**
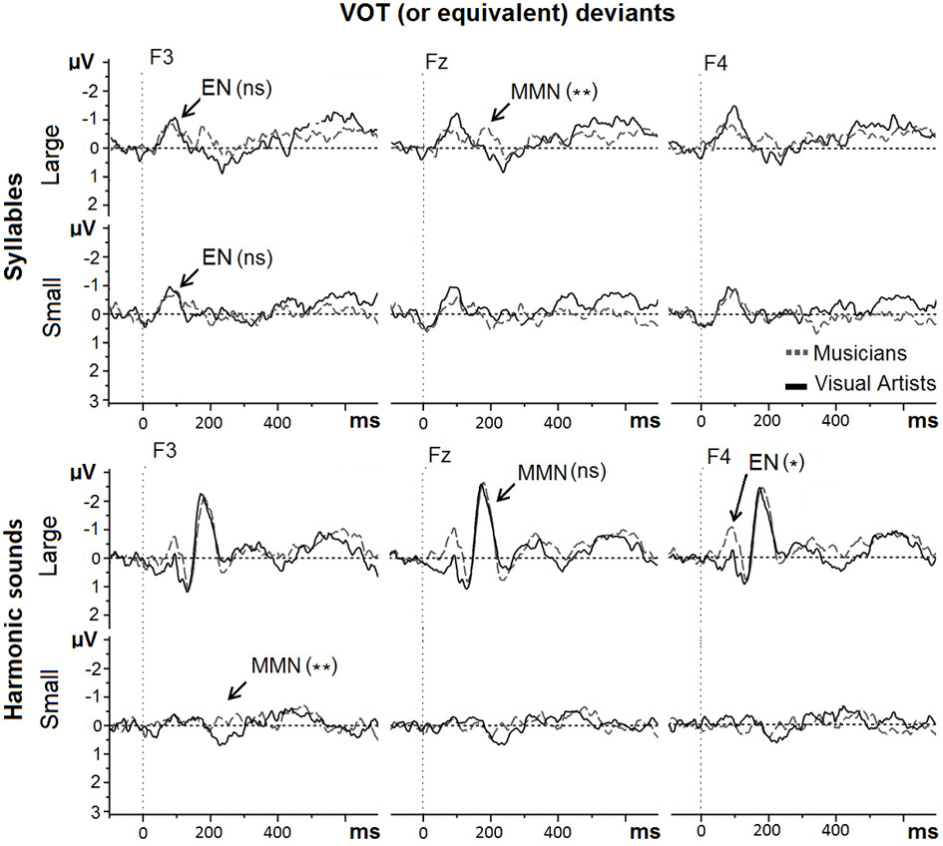
**MMNs to large and small VOT (or equivalent) deviants in Mandarin syllables and in harmonic sounds recorded at Frontal sites (F3, Fz, and F4) are overlapped for musicians (gray dashed line) and visual artists (black solid line)**.

For VOT deviants in Mandarin syllables (see Figure 5, upper row), the main effect of Group was significant [MUS = −1.31 μV; VA = −0.59 μV; *F*_(1, 42)_ = 7.77, *p* < 0.008]. Neither the main effect of Deviance size (Large deviant: −0.97 μV and Small deviants: −0.92 μV; *F* < 1) or the Group by Deviance size interaction were significant [*F*_(1, 42)_ = 1.27, *p* > 0.20]. To better understand the main effect of group, separate exploratory analyses were conducted for Large and Small VOT deviants. Results revealed that while the MMNs to Small VOT deviants were not significantly different in MUS (−1.14 μV) and in VA [−0.70 μV; *F*_(1, 42)_ = 1.33, *p* > 0.20], the MMNs to Large VOT deviants were significantly different between MUS (−1.48 μV) and VA [−0.47 μV; *F*_(1, 42)_ = 8.57, *p* < 0.006] thereby explaining that the main effect of Group was significant.

The early negativity (around 100 ms) to Large and Small VOT deviants in syllables was not different between groups (*F* < 1).

For the equivalent of VOT deviants in harmonic sounds (see Figure 5, lower row), the main effect of Group was significant [MUS = −2.01 μV; VA = −1.48 μV; *F*_(1, 42)_ = 4.47, *p* < 0.05]. Large deviants (−2.52 μV) elicited larger MMNs than Small deviants [−0.97 μV; main effect of Deviance Size: *F*_(1, 42)_ = 26.53, *p* < 0.001]. The Group by Deviance size interaction was not significant [*F*_(1, 42)_ = 1.23, *p* > 0.20]. Subsequent exploratory analyses revealed that while the MMNs to Large VOT-equivalent deviants were not significantly different in MUS (−2.62 μV) and in VA (−2.42 μV; *F* < 1), the MMNs to Small VOT-equivalent deviants were significantly larger in MUS (−1.40 μV) than in VA [−0.54 μV; *F*_(1, 42)_ = 8.87, *p* < 0.005] thereby explaining that the main effect of Group was significant.

For Large deviants, the early negativity (around 100 ms) was larger in MUS (−0.19 μV) than in VA [0.47 μV; *F*_(1, 42)_ = 4.72, *p* < 0.04].

## Discussion

Results are discussed in turn for each type of deviant in Mandarin syllables and in harmonic sounds before being considered together in a general discussion.

### Pitch contour deviants

One of the most interesting finding is that the MMNs to Small pitch contour deviants in Mandarin syllables were larger in musicians than in visual artists. Thus, in line with our hypothesis, musicians were more sensitive to small changes in the pitch contour of Mandarin syllables than visual artists. This conclusion is strengthened by the complementary finding that the MMNs to small pitch deviants were only significantly different from zero for musicians but not for visual artists. This is taken to show that while musicians automatically perceived the small difference in pitch with the standard syllable “Cha,” this difference was too small to be automatically detected by visual artists.

By contrast, no significant between-group difference in MMN amplitude was found for Large pitch contour deviants most likely because they were easy to perceive by both musicians and visual artists. In line with this conclusion the MMNs to Large pitch deviants were significantly different from zero in both groups. This is taken to show that the large pitch difference with the standard syllables was automatically detected in both groups. Importantly, these results extend to real Mandarin syllables previous results from Chandrasekaran et al. (2009) showing enhanced MMNs in English musicians than in English non-musicians to pitch contour deviants homologous to Mandarin tones. They also extend previous results from Kühnis et al. (2013) with pitch deviants in native vowels.

The finding that musicians are more sensitive to subtle pitch contour differences in Mandarin Chinese syllables than visual artists has interesting consequences for foreign language perception and learning. When immersed in a foreign language environment, automatic processing of subtle changes in pitch that are linguistically relevant (as in Mandarin Chinese) may largely contribute to the explicit learning of the language. Previous results have shown that musical expertise facilitates the attentive processing of pitch variations in a foreign language both at the segmental and supra-segmental levels (e.g., Marques et al., 2007; Marie et al., 2011b). However, to our knowledge the relationship between automatic and attentive processing has not yet been directly tested within the same participants. This may be an important aspect to explore in future experiments.

Results for harmonic tones are in line with many results in the literature showing larger MMNs to pitch deviants in pure tones, harmonic tones, and musical sounds in musicians than in non-musicians (e.g., Tervaniemi et al., 2001; Nikjeh et al., 2009; Marie et al., 2012). These results are typically interpreted as reflecting increased sensitivity to pitch contour in musicians than in non-musicians. Importantly, results for pitch contour deviants in harmonic tones were very similar to those reported above for Mandarin syllables. While the between-group difference was not significant for Large pitch contour deviants, that were easy to detect, the MMNs to Small pitch deviants in harmonic tones were larger in musicians than in visual artists. Thus, the effects of musical expertise are best seen when the differences between the deviants and the standard are difficult to perceive. However, and in contrast to Small pitch contour deviants in Mandarin syllables, for both types of deviants and in both groups, the MMNs were significantly different from zero so that both musicians and visual artists automatically detected Large and Small pitch deviants in harmonic sounds. Finally, the finding that musicians are more sensitive to Small pitch contour deviants both in harmonic sounds and in Mandarin syllables is in line with the hypothesis that speech and non-speech sounds rely on common pitch processing (Wong et al., 2007; Chandrasekaran et al., 2009; Kraus and Chandrasekaran, 2010; Besson et al., 2011; Bidelman et al., 2013).

### Duration deviants

Results were not as clear-cut for duration deviants in Mandarin syllables as for pitch contour deviants. As expected, the between-group differences were not significant for Large duration deviants that were easy to detect (120 ms shorter than the Standard) and the MMNs were significantly different from zero in both groups. However, in contrast to our hypothesis, the MMNs to Small duration deviants were not significantly different between musicians and non-musicians. It may be that the 40 ms difference with the Standard was too small to be automatically perceived. In line with this explanation, the MMNs to Small duration deviants were not significantly different from zero in either group. These results contrast with those reported by Milovanov et al. (2009) showing enhanced MMNs to speech duration deviants in 10- to 12-year-old children with high musical aptitudes and pronunciation skills compared with children who lacked these skills. Moreover, Chobert et al. (2011) also found larger MMNs to Large and Small duration deviants in 9-year old musician than in non-musician children. These different results are likely linked to the specific characteristics of the stimuli and the duration that was chosen for the Small duration deviants. In this respect, results of the pilot experiment showed that musicians were able to attentively detect the Small duration deviants better than non-musicians. Moreover, other results in active listening tasks point to enhanced processing of the metric structure of words presented in sentence contexts in musicians compared to non-musicians (Marie et al., 2011b) and to enhanced discrimination and identification of moraic features in musicians (Japanese and Dutch) than in non-musicians (Sadakata and Sekiayama, 2011). Thus, taken together, these results suggest that while the 40 ms duration difference between the standard and the Small duration syllabic deviants was possibly too small to be automatically processed, it could be attentively detected. Such dissociation between pre-attentive and attentive processing has already been reported in several experiments (e.g., Tervaniemi et al., 2009; Marie et al., 2012) and needs to be further explored.

For harmonic sounds, results were in line with our hypotheses with significant between-group differences for Small duration deviants and no significant group differences for Large duration deviants. Thus, the MMNs to large duration deviants were significantly different from zero in both groups, but not significantly different between musicians and visual artists, possibly for the same reasons as detailed above for Mandarin syllables: the 120 ms duration difference with the Standard was easy to detect and automatically processed by both musicians and visual artists. Moreover, an early negativity that seemed larger in musicians than in visual artists developed around 120 ms post-stimulus onset but this difference was not significant. By contrast, the P3a was clearly larger in musicians than in visual artists thereby showing that attention was automatically attracted to the large duration deviants in musicians but not in visual artists (Courchesne et al., 1975; Squires et al., 1975).

For Small duration deviants, the MMN was significantly different from zero in musicians but not in visual artists and the MMN was significantly larger in musicians than in visual artists. Thus, while musicians automatically processed the 40 ms difference in duration between the standard and the small duration deviants, this difference was too small to be automatically perceived by visual artists. These results are in line with those reported by Marie et al. (2012) showing enhanced MMNs to duration deviants in harmonic sounds in French musicians compared to French non-musicians. Importantly, however, these results also reveal differences in the processing of duration in syllables and harmonic sounds since the 40 ms difference with the standard was automatically perceived by musicians in harmonic sounds but not in syllables.

### Vot deviants

For Mandarin syllables, the syllable “Zha” was used as the Large VOT deviant (across phonemic category deviant). The finding of larger MMNs in musicians than in visual artists extends previous results related to the automatic processing of VOT in syllables belonging to the native phonemic repertory [Chobert et al. (2013) in children and Kühnis et al. (2013) in adults) to the automatic processing of VOT in foreign syllables. However, the MMNs to the syllable “Zha” were smaller and less-well defined as compared to those obtained for the syllables “Pa” (Chobert et al., 2013) or “Ta” (Kühnis et al., 2013). This probably reflects differences in acoustic features since the attack time for the syllable “Zha” is less marked than for stop consonants such as “P” and “T.”

By contrast, the MMNs to small VOT deviants in Mandarin syllables were not significantly different from zero in either groups and no significant differences were found between musicians and visual artists. As argued for Small duration deviants in syllables, the Small VOT deviants were probably too close from the standard to be automatically detected. These results contrast with those of the active listening task of the pilot study showing higher detection rates of Small VOT deviants in musicians than in non-musicians. Again, they point to some dissociation between automatic processing, as measured with the MMN, and attentive processing, as in the active listening tasks of the pilot study.

Finally, for harmonic sounds, the MMNs to large VOT-equivalent deviants were significantly different from zero in the two groups but not significantly different between musicians and visual artists. Thus, both musicians and visual artists were equally sensitive to the equivalent of a VOT manipulation, created by zeroing the first 60 ms of the harmonic tone. However, a fronto-central early negativity developed between 50 and 130 ms and was larger in musicians than in visual artists thereby indicating that musicians were more sensitive to this manipulation than visual artists. For Small VOT-equivalent deviants, MMNs were significantly different between the two groups. However, this result should be considered with caution because the MMNs were not significantly different from zero in either group which is taken to indicate that zeroing the first 30 ms of the harmonic sounds was too small of a difference from the standard to be automatically processed either by musicians or by visual artists. Again, the choice of the value for small deviants was based on the results of the pilot study showing that these stimuli were attentively detected with higher detection rates by musicians than by non-musicians. As proposed for Small duration deviants in syllables, these results suggest some dissociation between automatic and controlled listening when the deviant stimuli are close to the standard. Clearly, these results point to the importance of choosing the right stimuli to test for the effects of interest.

## Conclusions

Results for duration and VOT deviants were not clear-cut in showing similar effects of musical expertise for Mandarin syllables and harmonic tones, possibly due to the specific characteristics of the stimuli that were chosen based on results in an attentive listening task (pilot study). As such, they reveal interesting dissociations between automatic and controlled attentive processing that will be examined further by requiring participants to actively discriminate the different types of deviants. However, an alternative interpretation needs to be considered. The differences between the musicians and the visual artists tested in the present experiment may be smaller than between the musicians and the non-musicians tested in the pilot study for at least two reasons. First, the non-musicians of the pilot study had no strong artistic background (they were mainly Master and PhD students in Neuroscience). By contrast, the visual artists were professional artists with more than 7 years of intensive training. Thus, musicians and visual artists may have developed “artistic brains,” more similar to each other than to the “non-artistic brain” of the non-musicians tested in the pilot study. Second, visual artist typically spend 5–10h a day working on their creations and they listen to music most of the time while working. Thus, even if they did not receive formal music education, they may have developed a “musical ear” through thousands of hours of passive exposure to music. To test for this hypothesis, non-musicians only occasionally listening to music (i.e., scientists who are not music-lovers) should be used as a control group.

By contrast to results for duration and VOT deviants, results were clear-cut in showing that the processing advantage of musicians over visual artists for pitch contour deviants in harmonic sounds extended to pitch contour deviants in Mandarin syllables, specifically when the differences between the deviants and the standard are small. These findings are in line with the hypothesis that years of musical practice increase auditory processing abilities and confer an advantage to musicians not only for harmonic sounds but also for speech sounds (Kraus and Chandrasekaran, 2010; Strait et al., 2010; Besson et al., 2011). These abilities may turn out to be very important to facilitate the learning of foreign languages, specifically when pitch variations are linguistically relevant as in Mandarin Chinese and in many other languages of the world (e.g., most African languages).

An issue that has been hotly debated in the literature is whether the differences between musicians and non-musicians reflect genetic predispositions for music or are linked with extended musical training (e.g., Schellenberg, 2004; Hyde et al., 2009; Moreno et al., 2009; Corrigall et al., 2013). While genetic predispositions certainly play a role in the observed differences, Musacchia et al. (2008) showed, by using both sub-cortical and cortical measures, that processing the specific pitch features of the syllable “Ba” was correlated with the duration and the age of onset of musical training thereby pointing to the importance of musical training. Most importantly, results of longitudinal studies have demonstrated that effects similar to those found in cross-sectional studies comparing musician and non-musician children can be generated by training non-musician children with music (e.g., Moreno et al., 2009; Chobert et al., 2012; François et al., 2013). As these effects were found in children, it may be that there is a critical period for musical training so that different results would be obtained in adults trained with music. To our knowledge, such a study remains to be conducted to test for the hypothesis of a critical period for music learning.

Taken together, these results show that musical expertise positively influences the automatic processing of non-native supra-segmental contrasts. Musicians were more sensitive than visual artists to changes in syllabic pitch contours even if these changes did not belong to the phonemic repertory of their own language. These results raise the interesting possibility that, by being more sensitive to pitch and to lexical tone contrasts, musicians may learn tone languages more easily than non-musicians (e.g.,Wong and Perrachione, 2007). This hypothesis will be directly tested in future experiments.

### Conflict of interest statement

The authors declare that the research was conducted in the absence of any commercial or financial relationships that could be construed as a potential conflict of interest.

## Footnotes

^1^Note 1: This experiment also included an attentive listening condition (results not reported here) in which participants were asked to press a button to intensity deviants. As the intensity deviants were only included for this purpose, they are not analyzed in the present experiment.
